# Shades of gray: Conscientious objection in medical assistance in dying

**DOI:** 10.1111/nin.12308

**Published:** 2019-07-04

**Authors:** Barbara Pesut, Sally Thorne, Madeleine Greig

**Affiliations:** ^1^ School of Nursing University of British Columbia Okanagan British Columbia Canada; ^2^ School of Nursing University of British Columbia Vancouver British Columbia Canada

**Keywords:** assisted suicide, conscientious objection, euthanasia, medical assistance in dying, nurses, nursing ethics, nursing philosophy

## Abstract

With the advent of legalized medical assistance in dying [MAiD] in Canada in 2016, nursing is facing intriguing new ethical and theoretical challenges. Among them is the concept of conscientious objection, which was built into the legislation as a safeguard to protect the rights of healthcare workers who feel they cannot participate in something that feels morally or ethically wrong. In this paper, we consider the ethical complexity that characterizes nurses' participation in MAiD and propose strategies to support nurses' moral reflection and imagination as they seek to make sense of their decision to participate or not. Deconstructing the multiple and sometimes conflicting ethical and professional obligations inherent in nursing in such a context, we consider ways in which nurses can sustain their role as critically reflective moral agents within a context of a relational practice, serving the diverse needs of patients, families, and communities, as Canadian society continues to evolve within this new way of engaging with matters of living and dying.

## 1 INTRODUCTION

Imagine for a moment, you have a long‐standing relationship with a home‐care client who has decided to access medical assistance in dying (MAiD). In light of your relationship, the client asks you to help by assisting with planning and by being present throughout the process. What is your visceral response? Is it one of immediate agreement, honored that you would be asked to support? Or, does something inside you withdraw, knowing that despite your long‐standing relationship, you cannot become part of this act with which you so strongly disagree? Or, are you somewhere in the middle, unsure? In this paper, we discuss the ethical complexity that characterizes nurses' participation in MAiD and propose strategies to support nurses' moral reflection and imagination as they seek to make sense of their decision to participate or not.

In Canada, MAiD has become one end‐of‐life option alongside others. In February 2015, the Supreme Court of Canada released a landmark decision striking down the *Criminal Code's*' prohibition on Assisted Suicide (Carter v Canada, 2015). Sixteen months later, in June 2016, Bill C‐14: *An Act to Amend the Criminal Code and to Make Related Amendments to Other Acts* (Medical Assistance in Dying) (2016) received Royal Assent, thus formally introducing assisted suicide into Canadian legislation. Under *Bill C‐14*, medical assistance in dying (MAiD) is defined as follows: (a) the administration by a medical practitioner or nurse practitioner of a substance to a person, at their request, that causes their death; or (b) the prescribing or providing by a medical practitioner or nurse practitioner of a substance to a person, at their request, so that they may self‐administer the substance and in doing so cause their own death. To be eligible for medical assistance in dying, Canadian citizens must meet all of the following criteria: (a) They are eligible for health services funded by a government in Canada; (b) they are at least 18 years of age and capable of making decisions with respect to their health; (c) they have a grievous and irremediable medical condition; (d) they have made a voluntary request for medical assistance in dying that, in particular, was not made as a result of external pressure; and (e) they give informed consent to receive medical assistance in dying after having been informed of the means that are available to relieve their suffering, including palliative care. A grievous and irremedial medical condition is defined as a serious and incurable disease or disability which is in advanced state of irreversible decline and causes intolerable suffering and in which natural death is reasonably foreseeable (Bill C‐14).

Unlike the United States, where assisted death is legal but can only be self‐administered, or like Switzerland, where it is provided through parallel citizen‐based services (Ferguson, 2016), in Canada MAiD is integrated into the healthcare system. Health regions across Canada have formed multidisciplinary MAiD teams to support access to high‐quality care (e.g., Manitoba). Further, whether MAiD is self‐administered or clinician‐administered, in most health regions a physician or nurse practitioner is required to remain with the client until death. Registered nurses also perform important roles in MAiD such as participating in assessing clients for competency, providing information, coordinating the process including other healthcare personnel, preparing equipment and intravenous access, providing post‐death care, and supporting patients and significant others. As such, clinicians in Canada are an integral part of the MAiD process.

Despite this degree of healthcare integration, the issue of MAiD in Canadian society remains a deeply polarizing one (Wasylenko, 2017). Canadian media is rife with accounts of those championing MAiD and those opposed to MAiD. For example, a number of stories have profiled Canadians pursuing a ‘good death’ through MAiD and those who have assisted them in that pursuit (Grant, 2018). At the same time, other stories have highlighted the perceived abuses of the MAiD process (Roberts, 2018). Some of the roots of this polarization were evident in a recent discourse analysis of the Supreme Court of Canada's case in which the prohibition on assisted dying was lifted (Carter v Canada, 2015). In their analysis of the court documents from this trial, Beaman and Steele (2018) illustrated how a shift from religious to non‐religious perspectives had influenced judicial understandings of key concepts such as pain, suffering, and illness. However, these judicial understandings may not reflect the diversity of viewpoints about these concepts within Canadian society overall, as illustrated by the arguments put forward by interveners in the case (Beaman & Steele, 2018). There were over 25 interveners in the 2015 Carter v Canada case representing a broad range of perspectives in relation to MAiD. To respect this diversity of opinions, and the rights of healthcare providers in such a contentious landscape, the legislation guards the right of healthcare providers to conscientiously object to participation in MAiD.

Nurses who conscientiously object to participation have a professional obligation to inform their employers of that objection, to report requests for MAiD, and to not abandon their clients (Canadian Nurses Association, 2017a). Further, nurses are instructed to ensure that their choices are based on ‘informed, reflective choice and are not based on prejudice, fear or convenience’ (Canadian Nurses Association, 2017a, p. 37). The decision to conscientiously object cannot be arbitrary or based upon purely selective responses. Authors writing about MAiD within the nursing ethics literature have argued that this reflection is critical for professional integrity. For example, Zimbelman (1999) suggested that the nursing profession holds a place of power and status in society, and as such, nurses have a responsibility to reflect deeply on the good they aspire to do in light of that position. They need to be reflecting upon their own moral response to MAiD, but also upon the effect that participation in MAiD might have on nursings' contributions to the good of society more broadly. Likewise, McCabe (2007) cautioned nurses to think beyond the perspective of unmitigated autonomy when considering their position in relation to MAiD. Nurses cannot avoid moral reflection by simply arguing that whatever the patient wants is what the nurse should do. McCabe (2007) contended that such an approach puts nurses in the position of simply being service providers rather than professionals. She further suggested that to only consider the unmitigated autonomy of patients has the potential to make nurses into handmaidens of the law, similar to the historical problem of nurses being perceived as handmaidens to physicians. What is congruent throughout the literature is that nurses hold both an ethical and a professional responsibility to reflect carefully and critically about their decisions to participate, or not, in MAiD.

But how do nurses go about doing such reflection and what might be the factors that impact upon that reflection? A number of helpful resources have been created to assist nurses with reflecting on their decision to participate, or not, in MAiD (Canadian Nurses Association, 2017b; CLPNM, CRNM, & CRPNM, 2017). These resources typically present case scenarios and encourage nurses to reflect upon their responses to those scenarios while providing guidance for ethical behavior. However, it is important to recognize the factors that impact upon, and perhaps limit, this reflective process. First, moral philosophy recognizes the limitations of such independent and rational moral reflection, suggesting instead that, to some degree, our day‐to‐day moral behavior is determined by social norms and pressure. We tend to do what others around us think we should do even if we may disagree with it (Gaus, 2018). This was illustrated in a study from the Canadian province of Quebec in which nurses indicated that their intent to participate in MAiD, once it was legalized, was influenced by those with whom they worked (e.g., physicians) and their family (Lavoie et al., 2016). Second, studies have suggested that nurses may not understand what conscientious objection is, and guidelines provided through professional codes of ethics may not adequately address the clinical complexities faced by nurses (Lamb, Evans, Babenko‐Mould, Wong, & Kirkwood, 2017). Third, the endpoint of reflection is usually described as a binary in which the nurse chooses to be a conscientious objector or not across all situations and individual cases of MAiD. This is implied by the obligation of nurses to declare their positioning as a conscientious objector to their employers, preferably before they enter into a new employment contract (Canadian Nurses Association, 2017a). For some nurses, decisions about whether to participate in MAiD may be this simple. For other nurses, however, the decision may be more nuanced, contextual, and, indeed, gray. For example, a recent study of nurses' experiences with MAiD in the Canadian context illustrated that few nurses were strongly opposed or in favor of MAiD. Rather, ‘the majority of nurses shared stories of being in process, holding an in‐between space of uncertainty, reflection, and active sensemaking’ (Beuthin, Bruce & Scaia 2018, p. 515).

In this paper, we will grapple with some of that complexity. We begin from the assumption that in relation to MAiD, some moral discomfort and ambiguity is a hallmark of professional nursing. Grappling with that ambiguity through moral reflection, imagination, and deliberation may open nurses up to new perspectives. Further, a deeper understanding of the complexity of these issues gives nurses permission to view their participation in MAiD, or not, as a journey of learning that may be characterized by moral and ethical uncertainty. Therefore, in the remainder of this paper we will discuss this ethical complexity in relation to several factors that influence nurses' decisions to participate or not in MAiD including intuitional responses, relational impact, moral coherence with similar end‐of‐life decisions, and lastly, law and safeguards.

## 2 INTUITIONAL RESPONSES

Nurses' intuitional responses to participating in MAiD may be more important than one might first imagine. For example, in a recent study of nurses' conscientious objection, one participant stated, ‘my natural conscience would probably tell me not to kill somebody’ (Lamb, Evans, Babenko‐Mould, Wong, & Kirkwood, 2018, p. 5). In the nursing philosophical literature, arguments about the moral and ethical permissibility of MAiD have been largely categorized into arguments from intuition and arguments from philosophy. Snelling (2004) described these two types of arguments. Arguments from intuition are based within feeling ideas such as respecting autonomy or mercy, whereas arguments from philosophy are ‘more sophisticated arguments’ that involve ‘more detailed reflection about our intuitions’ (p. 352). In this case, philosophic reasoning is positioned as superior to intuition. A similar assumption about the hierarchy between intuition and rational argument undergirds much of the nursing ethics literature outside of the context of MAiD; nurses engage in rational moral reasoning as they seek to do what is right in the context of practice. For example, the Canadian Nurses Association's Code of Ethics provides a set of professional values, responsibilities, and a model for ethical decision‐making to help guide nurses through a reasoned process about what constitutes ‘safe, compassionate, competent and ethical care’ (2017a). From this perspective, a nurse can reflect upon the principles and values that undergird nursing practice and apply those principles to an act such as MAiD relatively unproblematically. It is a linear process of reasoning within a bounded act.

However, one suspects that coming to a conclusion upon the rightness of such an act through reasoning is intensely problematic, and indeed, that there are other more powerful influencers at work. For example, Haidt (2001, 2012) has explored a ‘social intuitionist’ model of moral reasoning. In this model, our moral judgements are quickly intuited, but we cannot necessarily explain that intuition. To make his point, Haidt explored a number of scenarios where it is difficult to justify why something is wrong, but one intuitively feels it to be wrong (e.g., cleaning the toilet with the flag). He identified these initial impressions as cognition rather than reasoning, and likened them to a form of perception. We perceive moral rightness, and this perception is constructed by sociocultural factors. From this perspective, much of our moral judgement is socially rather than individually determined, and as such, only changes under certain conditions. As such, there is a need to question whether reasoning is necessarily superior to moral intuition as Snelling (2004) has suggested. Perhaps there is something about the ways in which we interact with one another in community that critically inform our ways of being together in the world, ways in which we intuit rather than reason about moral goodness.

Other philosophers have suggested that this intuition may play important evolutionary roles over time. For example, Gaus (2018) proposed that what we intuit to be right has developed over an extended period of time and may play a more important societal role than what we can appreciate given our limited historical context. As humanity struggles collectively and incrementally with the problems of existence, we have no Archimedean point out of which to reason through how a particular moral position structures our relations and society. Thus, it is impossible to predict the consequences of dismantling that moral position. Indeed, Gaus (2018) suggested that we would be wise to break the moral rules carefully and in strategic ways so that we can appreciate the potential impact before we dismantle those rules full scale. One could speculate that the various ways in which societies around the world have chosen to enact assisted dying are indicative of this type of breaking the rules carefully. Canada chose the path of legislating MAiD which entailed constructing comprehensive rules and adopting specific obligations, the most important of which has been ensuring accessibility. Other countries have chosen the route of decriminalization without legislation. One could argue that this is a way of strategically evaluating impact without moving quickly to legislation. Evidence has suggested that nurses too practice this approach of breaking their moral rules cautiously to determine impact. Beuthin's et al. (2018) early study with Canadian nurses revealed a tentative approach toward MAiD that entailed uncertainty, reflection, and sensemaking.

If our moral intuitions are to some extent socially constructed, and constructed in such a way that they play important evolutionary roles, one can appreciate why Haidt (2001, 2012) further proposed that these moral intuitions are rarely challenged unless there is significant reason to do so. Indeed, he suggested that there are powerful motivations to stay with our moral intuitions, as they ensure that we remain coherent within ourselves and harmonious with those around us. However, in a morally conflicting situation, we still feel it is necessary to provide reasons for our intuition and so we may engage in a particular form of pseudo‐reasoning based upon posthoc justification. In such lines of reflection, we start from our moral intuition and reason backward, using only those arguments that justify our original intuition. In the relatively rare situations where we do change our moral intuition, it is usually accomplished through the reasoned logic characteristic of skilled philosophers, or through reflection in which we take on the roles of others and imagine their responses and subsequent dilemmas. Therefore, moral intuition is more likely to be changed where there is significant social interaction around a phenomenon.

This social intuitionist model has important implications for how nurses reflect on, and talk about, their willingness to participate in MAiD. It is not enough to recommend that nurses sit down with a set of principles and reason their way through the rightness or wrongness of this act, and hence, their willingness to participate. Rather, nurses need to engage both in a critical reflective process of reasoning and in conversations with others about how they are making sense of their moral positioning. Moral intuition and moral reasoning are important and complementary approaches.

This critical reflective process can begin with the identification of, and reflection upon, our own initial moral intuitions. We can further examine our tendency toward pseudo‐reasoning by searching for those ways in which we only seek to justify our decision to participate, or not, in MAiD. Instead, we should be searching for the reasons that would support alternative positions, reasons that we have not yet considered. If change can happen through reflective imagination, then it is incumbent upon us to imagine the world of those who choose MAiD, to imagine what it might be like to suffer with no hope of remediation of that suffering.

This process of reflective reasoning can be greatly enhanced through conversations with those whose opinions and experiences are different than our own. This can be difficult in a field as contentious as MAiD. However, if our morality is to some extent socially constructed, then it is arguable that we ought to consider engaging in conversations with others whose moral intuitions may reflect a departure from our own in order to be confident that our positions have the benefit of being informed by critical reflection. If morality is indeed an evolutionary process, then as Gaus (2018) suggested, the fact that some nurses choose to participate in MAiD, while others choose to conscientiously object, is, ultimately, a good thing. We can learn from one another as some hold firm to conscientious objection, others participate fully, and yet others engage tentatively to varying degrees as they learn more of the potential impact. Such diversity allows us to strategically engage this new moral landscape, all the while observing the consequences of our choice.

## 3 RELATIONAL ETHICS

If moral judgement is largely socially influenced (Haidt, 2001, 2012), then the effect of participating in MAiD on ones' relationships is an important consideration for nurses. For instance, what effect might a decision to participate, or not, in MAiD have on nurses' relationships with patients, on nurses' relationships with their own significant others, and on nurses' relationships within the broader community?

Nurses' relationships with patients are described in the literature as one of genuine caring and mutual respect (Ferrell & Rivera, 1995); of experiencing patients as persons (Davis, 1994); and of respecting and protecting life (Kowalski, 1993; Sullivan, 1999). Wurzbach (2000), in a discussion of the nursing role in euthanasia, suggested that the defining ethic of care is that nurses are the ones who ‘stay with’ (p. 117) patients. Staying with is characterized by such things as presence, active engagement, being nonjudgemental, and interpreting the dying process for patients and families. She suggested that this ‘staying with’ (Wurzbach, 2000, p. 117) may be the most important aspect of care, because dying clients need to believe that they will not be abandoned. How nurses think about this relationship of care is an important consideration when reflecting on conscientious objection. Is it a relationship of care in which nurses covenant to stay with patients regardless of their agreement or disagreement with the decisions patients make about their own care? Or, is it a contractual relationship, something to be negotiated depending upon the conditions that present themselves in the context of the relationship? If it is a contract of care, what are the choices patients make that will breach that contract? Is choosing MAiD one of those contract‐breaking choices?

This relationship of care is also informed by how nurses choose to negotiate diverse values and beliefs in the context of a professional relationship. When clinical choices that reflect antithetical values and beliefs arise, the nurse must then decide whose values and beliefs are preeminent in the context of the nurse–patient relationship. If the patient believes that MAiD is morally acceptable but the nurse does not, whose choice trumps? Or, are there ways to hold both choices concurrently? Although nurses are permitted to conscientiously object to participating in MAiD, how they convey that objection to clients is of paramount importance. As professionals, nurses are required to withdraw from care in such a way that their conscientious objection does not negatively impact their clients by communicating judgement or disapproval. Nurses must remain deeply concerned about the potential impact of their own values and beliefs on clients.

Beyond considerations inherent in the nurse–patient relationship, nurses also need to consider the other constellations of relationships they hold and honor. This is particularly important when evidence has indicated that these relationships are important factors in nurses' intent to participate in assisted death (Lavoie et al., 2016). It is not uncommon to meet nurses involved in MAiD who indicate that they engage in the procedure secretly, apart from the knowledge of their friends and family. As one nurse mentioned to me (BP) recently, ‘my parents are strongly against MAiD, and they would be terribly disappointed to know that I am involved. So, I just don't tell them’. This nurse is exercising moral agency in a way that could disrupt relationships with family. Others, however, might decide that this cost is simply too high. Thus, if morality is not just an individualistic, rational endeavor, but rather a socially embedded and contextually relevant construct, then these social relationships should be expected to play a key role. Indeed, MAiD may be a somewhat unique situation in nursing practice. For example, in most nursing situations, outside social relationships might have little bearing on the professional relationship between a nurse and a patient. However, in the context of MAiD whereby professionals are variously viewed as compassionate healers or killers depending upon ones' perspective, social relationships may play a stronger role in the context of the nurse–patient professional relationship.

Another context to consider is the nurses' relationships within the broader community. For example, in rural communities nurses' personal and professional roles are inextricably linked (Pesut, Bottorff, & Robinson, 2011; Pesut, Robinson, & Bottorff, 2013). These dual relationships mean that what nurses do in their professional lives has a profound impact on their personal relationships and vice versa. This dynamic can support high degrees of professional accountability for care. Nurses who care well often have special status within their community. However, the reverse can also be true. Choosing to participate in MAiD in rural communities may have a negative impact on the nurse's reputation in that community. Community members who strongly oppose MAiD may be unwilling to receive services from a nurse who they know has participated in MAiD. For rural nurses, the choice may be a lose‐lose scenario, particularly in terms of patient accessibility to care (Schiller, 2017). Nurses who choose not to participate may be limiting access to MAiD; nurses who choose to participate may reduce accessibility to regular care for those patients who are frightened to see a MAiD provider. Both choices have implications for colleagues. Rural physicians find themselves in a similar situation. Collins and Leier questioned whether there is a special obligation for rural physicians to provide MAiD because of their unique circumstances and wondered about the potential impact of being the ‘only game in town’ (2017, p. 189). If patients refuse to see a MAiD provider, then they must be cared for by other professionals in the community, and in rural communities, there is often a shortage of care providers.

What these scenarios illustrate is the profoundly social nature of the decision to be a conscientious objector or not. In clinical encounters with individual patients, a nurse who chooses to not participate in the MAiD process may experience the moral effects of failing to abide by the ethic of ‘staying with’ (Wurzbach, 2000, p. 117) patients. But, it may be no less distressing for a nurse to ‘stay with’ a patient when it violates his or her deeply held values and beliefs. Such decisions are socially embedded and socially impactful. Assisting nurses to identify and reflect upon these relational responsibilities in the context of MAiD may be one way to help attenuate this impact. Although philosophizing about the nature and substance of the nursing discipline has received less attention in an era of evidence‐based practice, momentous practice changes, like MAiD, should drive us once again to considering the nature of our discipline. Nurses, particularly during their formative educational years, can be encouraged to delve deeply into those disciplinary questions that would help them to make sense of interventions such as MAiD. What are the scope and boundaries of their commitments to patients? Is there an agreed‐upon endpoint of practice, and if so, what will that be? Such critical reflections can assist nurses early on to develop habits of thinking that will support moral development over a lifetime of nursing practice that may present new moral options that up until that point were unimaginable.

## 4 MORAL COHERENCE

Moral coherence is also an important motivator for developing moral judgement (Haidt, 2001). When confronted with a new moral angle, or a change in practice that conflicts with our moral stance, there is an instinctive desire to make sense of it in a way that is coherent with who we are as individuals. We want to know that our moral decisions are not haphazard, but something that we have thought through carefully and applied consistently. For example, think about the nurse whose faith commitment provides a moral intuition that MAiD is wrong based upon the sanctity of life. Unless the nurse can find other principles out of his faith tradition to support MAiD (e.g., free will or compassion), he must conscientiously object to participating in MAiD or otherwise risk moral incoherence.

Coming to moral coherence can be supported by considering similar situations in which coherence has already been achieved. For nurses, the clinical situations that are most closely related to MAiD are end‐of‐life decisions that also have the potential to hasten death. These include the decision to actively withhold treatment (e.g., antibiotics for frail elderly persons with pneumonia), the decision to withdraw or withhold life necessities (e.g., food and fluid), or the decision to give large doses of medication necessary to alleviate intense pain or suffering (i.e., that may inadvertently hasten death). If the nurse is morally comfortable with these treatments that have the potential to hasten death, but does not feel comfortable with MAiD, then to be morally consistent, the nurse may need to wrestle with the similarities and differences across these treatments. For example, some of that wrestling may be related to the framing of the intent; is the act intended to ‘kill’ (i.e., cruelty), or is it intended to support (i.e., comfort)? Two questions, derived from the literature on nursing ethics and MAiD (e.g., Begley, 1998; Gauthier & Swigart, 2003; Rich & Butts, 2004), can help to inform that wrestling. First, is there a distinction between killing and letting die when both actions lead to a hastened death? And second, should nurses reach their moral decisions purely through the application of principles (e.g., sanctity of life), or is there room for the consideration of outcomes (e.g., alleviation of suffering)?

A common argument against MAiD is that there is a morally significant difference between withholding or withdrawing a treatment that will lead to death and administering a medication that will lead to death (e.g., Goodman, 1996). However, in testing the soundness of this position, ethicists have constructed examples that challenge perceived differences in the moral culpability between someone who intentionally kills another and someone who stands by and simply lets another die, even when it is within their power to assist. Some would argue that both individuals are morally culpable for the subsequent death; one cannot differentiate culpability when the foreseeable outcome is the same (e.g., Begley, 1998; Dines, 1995). If that is the case, then nurses must search for reasonable grounds upon which to establish coherence between their decisions to be involved in other actions that may hasten death but not MAiD.

The second question of whether a nurse can adhere to principles without also considering consequences is particularly relevant for those who conscientiously object to MAiD based solely upon rigorously held deontological principles. Snelling (2004) identified this as the tension between deontological and consequentialist ethics and argued that in no ethical situations do nurses draw exclusively on one to the neglect of the other. Nursing practice is by nature outcome based; nursing interventions are designed based upon a preferred outcome. Therefore, coherence requires nurses to reflect on both principles and consequences in morally complex situations. For example, those who appeal to religious ethics often do so on the basis of moral laws that underpin goodness in the world (e.g., thou shalt not kill, obey your parents). Although these laws are important considerations, they cannot be applied indiscriminately or without contextualization (Fowler, 2009). Religious people can, and do, negotiate these principles on a daily basis, making judgements about their applications to a particular context (e.g., thou shalt not kill in the context of protecting other life). As nurses think about the principles that inform their responses to MAiD, it is also helpful to think about the outcomes to which they aspire as they provide care. A nurse who conscientiously objects to MAiD has a professional obligation to do so after having carefully thought through both the principles upon which they are making that decision (e.g., do not harm, sanctity of life) and the potential consequences for themselves, their patients, and society.

As nurses seek for moral coherence across end‐of‐life decision‐making, a useful strategy entails reflecting on questions that illuminate both the underlying principles and outcomes that inform these positions. For example, a case study in which various strategies can be used to attenuate suffering, but in which the outcome is always imminent death, could assist nurses to identify morally troubling questions inherent in each approach. Grappling with these questions in ever deepening ways may support nurses, and the profession overall, to respond to morally complex situations out of a coherent ethical foundation.

## 5 LAW AND SAFEGUARDS

To this point, we have discussed intuitional responses, relational ethics, and moral coherence as important factors for nurses to consider when deciding whether or not to participate in MAiD. However, there are additional, somewhat unique, circumstances in Canada that may serve to further complicate the decision. The legislative language that guides the implementation of MAiD in Canada is unclear, which leaves room for variable clinician interpretation of who is eligible for MAiD. In a comprehensive report on the challenges of interpreting Canada's MAiD legislation, Downie and Chandler (2018) expounded on six key phrases from the legislation that are particularly difficult to interpret. In this paper, we will discuss the three that are most relevant for nursing practice. The first is that ‘natural death has become reasonably foreseeable’ (Downie & Chandler, 2018. p. 5). Downie and Chandler suggested this phrase could be interpreted to mean that the death will happen naturally, or that it will happen within a particular timeframe, or that it will happen related to some identifiable cause. The concept of timing is further complicated by what foreseeable means (e.g., days as opposed to years). The second is that the patient must have a ‘serious and incurable condition’ (Downie & Chandler, 2018, p. 16). Here, Downie and Chandler grappled with whether incurable is defined in medical terms (any chance of a cure—even remote), or whether it was patient defined (e.g., cure from a treatment which is acceptable to the patient). The third is the requirement of ‘suffering that is intolerable to them’ (Downie & Chandler, 2018. p. 9). Specifically, Downie and Chandler wondered ‘does it mean suffering that literally cannot be tolerated, or does it mean an intensity of suffering that is at the far end of a spectrum running from mild to extreme?’ (2018, p. 19).

Downie and Scallion (Forthcoming), in a legal analysis of the language of *reasonably foreseeable natural death*, argued that ‘there is no temporal proximity limit on eligibility for access to MAiD in Canada’ (p. 30). In other words, length of prognosis is not relevant. Rather, natural death becomes reasonably foreseeable when ‘in the professional opinion of the medical or nurse practitioner, taking into account all of the patient's medical circumstances, how or when the patient's natural death will occur is reasonably predictable’ (Downie & Scallion, Forthcoming, p. 30). They provided practical examples of who would be eligible and not eligible. For example, a patient with Parkinson's or intractable anorexia would be eligible. A ‘40‐year old patient with incurable cancer for which suffering can be controlled by means acceptable to the patient’ (Downie & Scallion, Forthcoming, p. 31) would not be eligible.

Nurses who choose to participate in MAiD are encouraged to ensure that the safeguards laid out in the MAiD legislation are met prior to taking part (e.g., College & Association of Registered Nurses of Alberta, 2017). These safeguards are meant to ensure that each MAiD case is approached, implemented, and documented with careful diligence to a variety of factors meant to protect both patients and practitioners. The process is meticulously laid out and health regions across the country have created best practice policies, procedures, and practice supports. However, this meticulous attention to detail is potentially undermined by the vague and uncertain language contained in the legislative safeguards about who is eligible for MAiD. The degree of clinician interpretation required for eligibility for MAiD in Canada has important implications for nurses. Even if nurses are morally certain about their decision to participate, they may find themselves legally uncertain about whether the patient they are caring for does meet the criteria according to the legislation. This would in turn have moral implications. Further, if nurses' moral imagination and reflection has occurred within specific clinical scenarios, such as imminent death, they may need to reflect on how that reasoning holds up under a different scenario. The process of providing MAiD to a cognitively alert person with Parkinson's may feel morally quite different than providing MAiD to a person experiencing intractable suffering during the final days of life. These differing situations may present a moral context within which nurses have little experience.

Given the vagueness of language in the legislation, we can anticipate that the clinical presentations of those receiving MAiD will vary in accordance with the interpretation of the clinicians who act as MAiD assessors. Therefore, when considering their participation, or not, in MAiD, nurses should also consider these variable presentations and grapple with their own interpretations according to the spirit and the letter of the law. It is important to qualify here that this is not to be perceived as nurses being responsible for these clinical decisions; that responsibility remains with MAiD assessors and providers (i.e., physicians and nurse practitioners). However, should nurses choose to participate, they need to be satisfied within their own clinical judgement that the safeguards have been met and within their own moral judgement that this is a decision in which they can take part. To participate in MAiD without that moral deliberation is to invite moral distress. (For a more fulsome discussion of nurses' moral experiences see Elmore, Wright, & Paradis, 2016) Further, case presentations designed to assist nurses to develop their moral reasoning in relation to MAiD should pay careful attention to this variability that nurses will inevitably encounter in practice. As a result of such variability, we may expect that each nurse may not be able to self‐identify in black and white terms as conscientious objector or not, but rather as one who encounters each unique situation, and from that complexity, carefully crafts a moral response.

## 6 CONCLUSION

For some nurses, the decision to participate, or not, in MAiD will be relatively simple, and for others, it will be complex and uncertain. In this paper, we have proposed a number of factors that may influence the decision to participate or not, and a number of strategies nurses can use as a starting point for moral imagination and deliberation. Discerning ones' moral intuition, reflecting on that intuition, and engaging in conversations with others of differing viewpoints, seems a good starting point. From there, nurses can explore and weigh the impact of their decision on the constellation of their personal and professional relationships. Grappling with questions about similar end‐of‐life decisions that also hasten death may support some degree of nurses' moral coherence. However, the diverse clinical conditions of patients who are eligible for MAiD may mean that nurses are on unfamiliar moral ground. This may make it difficult for nurses to clearly define themselves as conscientious objectors or not. Instead, they may find themselves on a path of moral discovery whereby they need to engage in and reflect on the issues, both as individuals and in conversation with others. Dwelling in this gray zone of moral learning may be uncomfortable for nurses and inconvenient for a system that requires nurses to identify as conscientious objectors or not. But, in that discomfort we would do well to remind ourselves that nurses have a long and distinguished history of crafting and evolving coherent, and increasingly robust, moral responses to this complex healthcare world.
